# *Pasteurella multocida* infection: a differential retrospective study of 482 cases of *P. multocida* infection in patient of different ages

**DOI:** 10.1186/s12879-025-10711-1

**Published:** 2025-03-04

**Authors:** Bo Wei, Chang Liu, Jie Zhu, XinYu Zou, Zhenhua Zhang

**Affiliations:** 1 Department of Infectious Diseases, The Second Affiliated Hospital of Anhui Medical University, Hefei, 230601 China; 2 Department of Pediatrics, The Second Affiliated Hospital of Anhui Medical University, Hefei, 230601 China

**Keywords:** *Pasteurella multocida*, Age, Clinical characteristics, Antibiotic sensitivity

## Abstract

**Background:**

There is limited data regarding the sources of human *Pasteurella multocida* infection, characteristics of infected populations, and the antibiotic resistance patterns of human strains.

**Methods:**

Through the multi-database platforms, we conducted a comprehensive review and analysis of 482 reported cases of *Pasteurella multocida* from the world since from 1964 to 2023, including the sources of infection, sex and age distribution of infected individuals, and the clinical manifestations of infection in different age groups. Additionally, we evaluated the antibiotic susceptibility of different strains of the bacterium.

**Results:**

*P. multocida* infection is mainly involved in infants and the elderly population, and it is closely related to animal exposure (cats 54.1%, dogs 29%), especially cat-related infections in adults and older are significantly more common than in children (*P* = 0.005, *P* < 0.001). Infection with *P. multocida* can cause local redness of the skin and soft tissue (11.6%), and also progress to systemic infection, like central nervous system (14.5%), especially in children, Cardiovascular system (29.3%), respiratory system (21.4%), digestive system (12.9%), urogenital system (2.9%) and bone and joint infections (5%). In terms of treatment, first-line treatment is priority to with penicillin. However there are also resistance to *Penicillins* and β-lactam antibiotics (18 cases were reported), and strains derived from wounds, blood and respiratory tract are resistant to multiple antibiotics.

**Conclusion:**

*P. multocida* primarily causes infections through cats and dogs in different age groups, leading to various clinical manifestations and outcomes. It is generally sensitive to penicillin antibiotics but exhibits varying resistance among strains of different clinical origins. Studying these aspects is crucial to raise awareness about preventing *P. multocida* infections and to standardize clinical treatment approaches.

**Supplementary Information:**

The online version contains supplementary material available at 10.1186/s12879-025-10711-1.

## Introduction

*Pasteurella multocida (P. multocida)* is an anaerobic, gram-negative coccobacillus, a species of the family *Pasteutellaceae*. *P. multocida* was first discovered by Perroncito in 1878 and named after Louis Pasteurella; It was first isolated in 1880 and was proposed to be one of the pathogens responsible for poultry diseases. *P. multocida* is commonly found in the oral, nasal and respiratory cavities of animals (such as cats or dogs), but also in domestic cattle, rabbits, pigs, birds and a variety of wild animals. The carriage rate of *P. multocida* isolated from the oropharynx of animals was 70%−90% and 20%−50% in cats and dogs respectively [1]. In animals, this bacterium is a well-known cause of various conditions, including avian cholera in birds, hemorrhagic septicemia in ruminants, and respiratory diseases in cattle. It is also responsible for progressive atrophic rhinitis and pulmonary pasteurellosis in pigs, etc. [2, 3].

Secondly, *P. multocida* is also a rare zoonotic pathogen. At present, the research on *P. multocida* infection is relatively rare. The existing research literature mainly focuses on case reports, which found that *P. multocida* infection in human beings is mainly caused by animal biting, scratching or licking and contact with nasopharyngeal secretions [4, 5]. In the United States, about 300,000 patients appear to emergency rooms annually due to animal bites or scratches [6]; And studies on animal carrier rates, a bacterial isolate of infected wounds of 50 dog bites and 57 cat bites identified *Pasteurella* as the most common isolate in dog bites (50%) and cat bites (75%); *Pasteurella canis* was the most common isolate of dog bites, and *P. multocida* subspecies multocida and septica were the most common isolates of cat bites. However, its clinical manifestations of human infection are relatively rare [7]. *P. multocida* infection in humans often presents with local skin soft tissue wounds, in addition, it also can progress to systemic infection. Local wound infection is mainly manifested as redness, pain, suppuration and other symptoms, and even will appear necrosis and ulcers. In addition, most systemic infected patients can be high fever, chills, palpitations, shortness of breath, which can lead to organ failure, shock or death. The clinical symptoms of *P. multocida* infection are often not specific and easily overlooked, moreover, due to individual differences, different populations may have different clinical features. So, based on the existing case reports of *P. multocida*, we grouped infected patients according to age, we classified and analyzed the patients, so as to provide a new and more reliable reference basis for understanding the infection characteristics of *P. multocida* in different age groups, and for the clinical diagnosis and treatment of *P. multocida*.

For the treatment of patients with *P. multocida* infection, empirical treatment with penicillin antibiotics is adopted in clinical practice. As for the study of antibiotic susceptibility of *P. multocida*, most literature only describe the susceptibility results of cultured strains in each case, and larger-scale studies are lacking, therefore, we also explored the antibiotic sensitivity of *P. multocida* and the antibiotic resistance in different clinical specimens.

## Materials and methods

### Population basic data acquisition and inclusion criteria

**Fig. 1:**
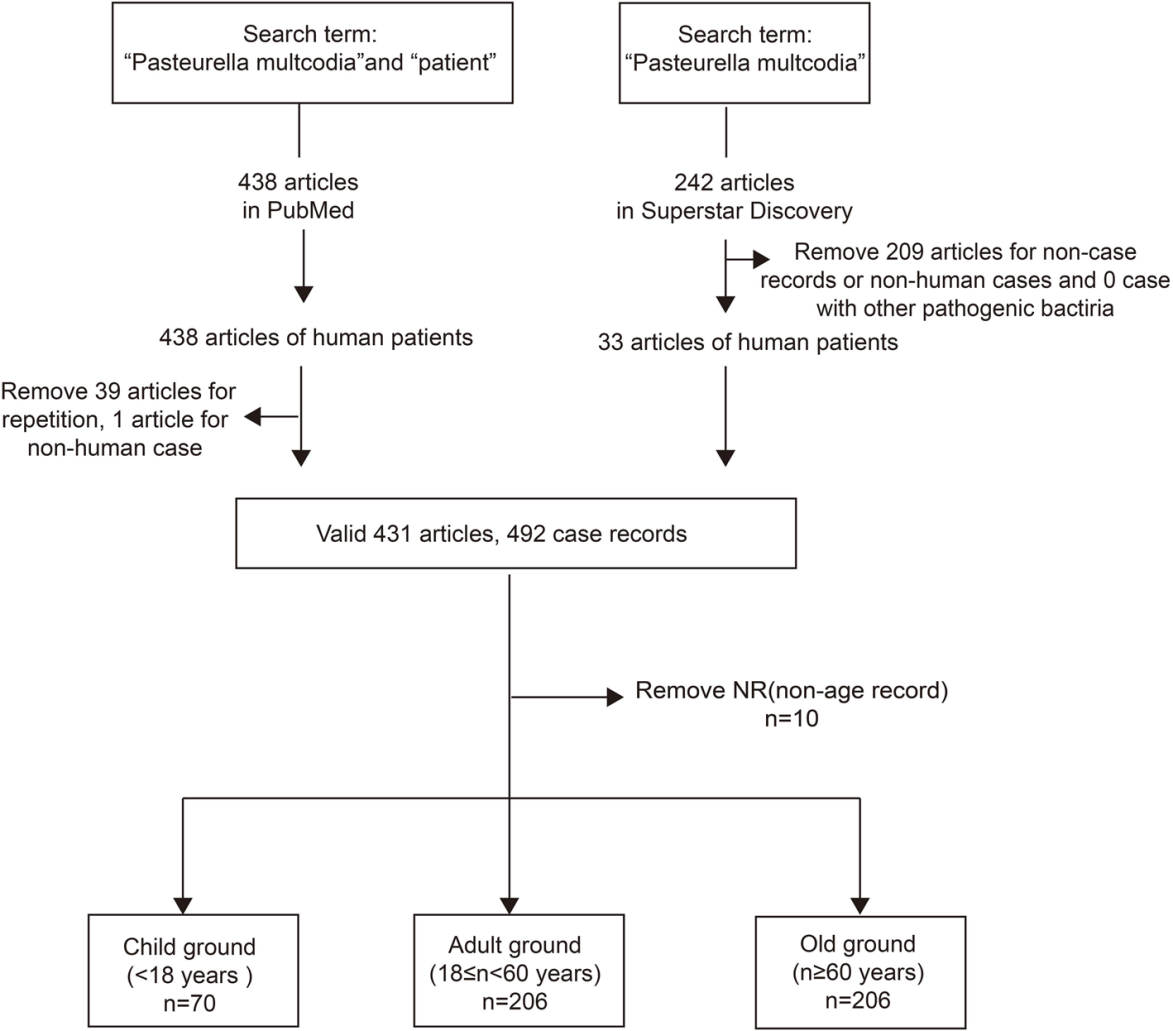
Study data acquisition and grouping methods

The overall workflow of the study is shown in Fig. 1. To conduct this research, we have searched for articles related to “Pasteurella multocida” and date (1964–2023) in Chinese on Superstar Discovery database (https://ss.zhizhen.com/) and “(Pasteurella multocida) AND (patient) AND (Filters: from 1964/1/1–2023/1/1)” on PubMed database (https://pubmed.ncbi.nlm.nih.gov/). Basic information of patients, infection route, clinical manifestations, drug susceptibility test results, prognosis and other information of patients in 431 literature cases, and a total of 492 cases were reported. After removing 10 cases where patient age was not recorded, there were left with 482 cases of infection, which we divided into three groups: children (under 18 years old), adults (18 to 60 years old), and the elderly (over 60 years old).

The inclusion criteria were: 1) Cases with a clear age of the patients, and 2) Human cases of *P. multocida* infection. The exclusion criteria were: 1) Cases of non-human infection with *P. multocida*, 2) Combined cases with the presence of infections with other pathogenic bacteria, and 3) Duplicate reported cases.

### Origin and route of infection

To understand the source and transmission routes of *P. multocida*, we grouped the collected sources of infection into animal-derived and non-animal-derived cases. For cases with animal-derived infections, we categorized and counted the specific animal species involved. Additionally, we summarized the modes of transmission, which included traumatic infections like bites and scratches, and the specific sites of infection, such as the face, trunk, etc. Finally, we analyzed the gathered statistics across the different groups, and also separately counted the cases with unclear sources of infection.

### Clinical manifestation

In relation to the clinical presentations of *P. multocida* infection, the cases were categorized by the affected systems: central nervous system, cardiovascular system, oropharyngeal and respiratory tract, digestive system, skin and soft tissue, bones and joints, genitourinary system, and eyes. Additionally, data on the incubation period and clinical outcomes of the infection were gathered, with clinical outcomes classified into three categories: full recovery, poor prognosis, or death. This information was analyzed to understand the distribution and differential characteristics within each group.

### Antibiotic options for the treatment of *P. multocida*

In order to better understand the antibiotic susceptibility characteristics of *P. multocida* and to explore whether drug susceptibility differ in different site strains, We categorized the recorded antibiotic susceptibility results for each case into three groups: sensitive, intermediate, and resistant. We then compared the differences in antibiotic sensitivity across these categories and compared the antibiotic resistance of the derived strains at various infection sites.

### Statistical analysis

All data collected in this study were processed by using IBM SPSS Statistics 26.0. Count data of age, sex, source of infected animals, infection mode, clinical characteristics, etiology results and treatment outcome were expressed as cases and percentage (%), using *χ*^2^ test for comparison between groups; *P* < 0.05 was statistically significant, and *P* < 0.01 was statistically significant. For each antibiotic class (*Penicillin*, C*ephalosporins*…), sensitivity effect (grade data), sensitivity (S) = 1, medium (I) = 0, resistance (R) = −1, rank sum test. For the three indicators in {S, I and R}, if there is one indicator with data (non-NR), the whole {S, I and R} data will be included in the test. For {S, I, R} of the included tests, N was considered if the index is NR. Finally, the complete sample size is *N* = 243, and statistical analysis. The same data was processed and analyzed for unrecorded data. A visual presentation of the obtained results was applied by GraphPad Prism 8.

## Result

### Basic situation of patients infected with *P. multocida* infection

**Fig. 2:**
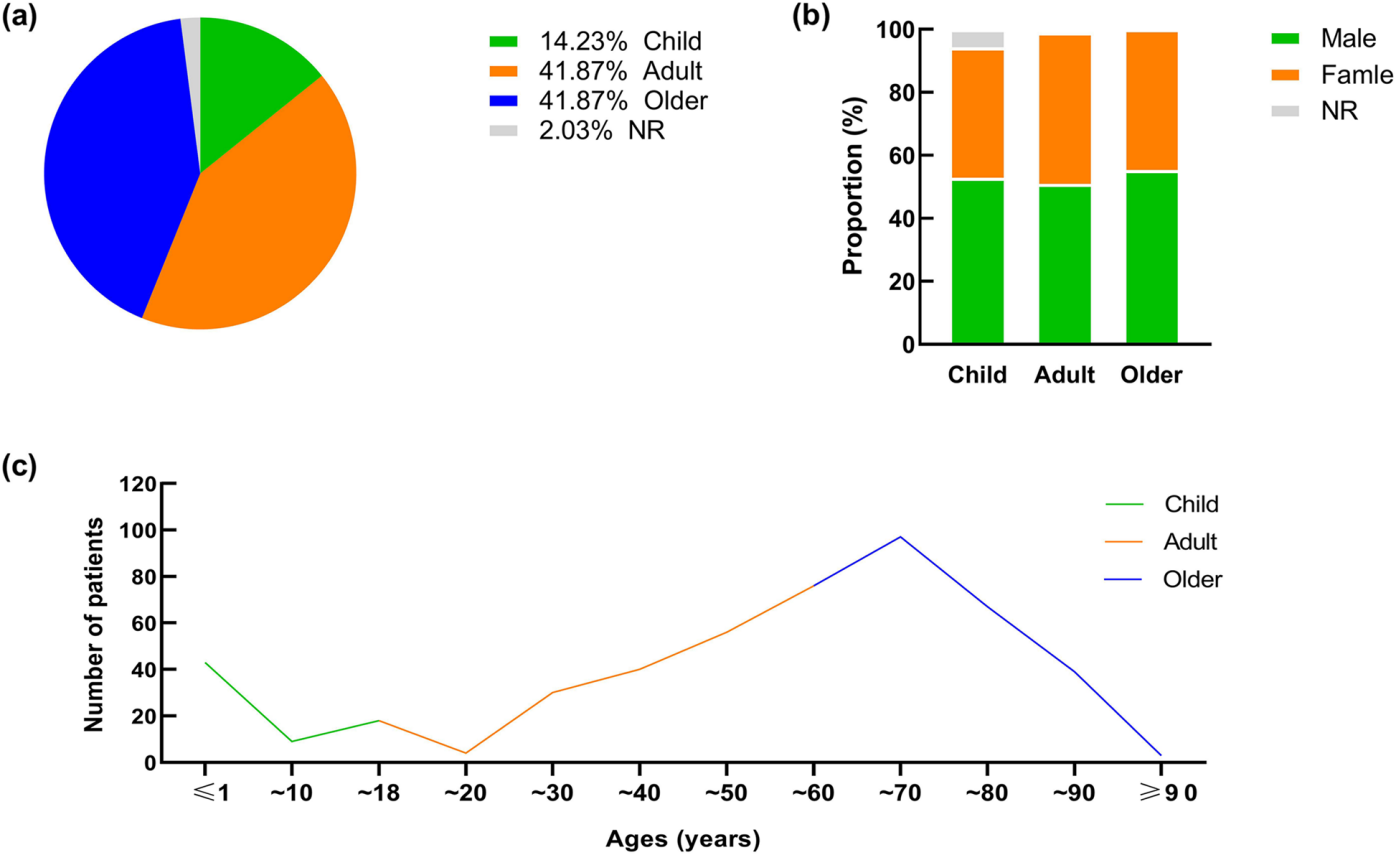
Characteristics of patients with *P. multocida* infection. **a** Number and proportion of patients in each group; **b** Gender ratio within three groups; **c** Trends in incidence rates across all age groups

We have recorded 492 cases since from 1964 to 2023, of these, 459 cases were probably from Europe and America, and 33 cases were from Asia. We grouped the cases according to the age, children 70 cases (14.23%), adults and the elder both 206 cases other than these 10 cases. Among the age and sex of those infected cases (41.87%), and 10 cases had no documented age (Fig. 2a). Then, we study all with *P. multocida*, males are predominant, about 53.8%. But in *P. multocida*-infected populations, both sexes are susceptible and show no significant difference (Differences between the three groups, all *P* > 0.05 (Fig. 2b). Regarding the trend in age at onset, we found that children and the elderly were the peek in the age of onset of the infection and the number of infections increased with age (Fig. 2c).

### The main route of infection of *P. multocida* infection

**Fig. 3:**
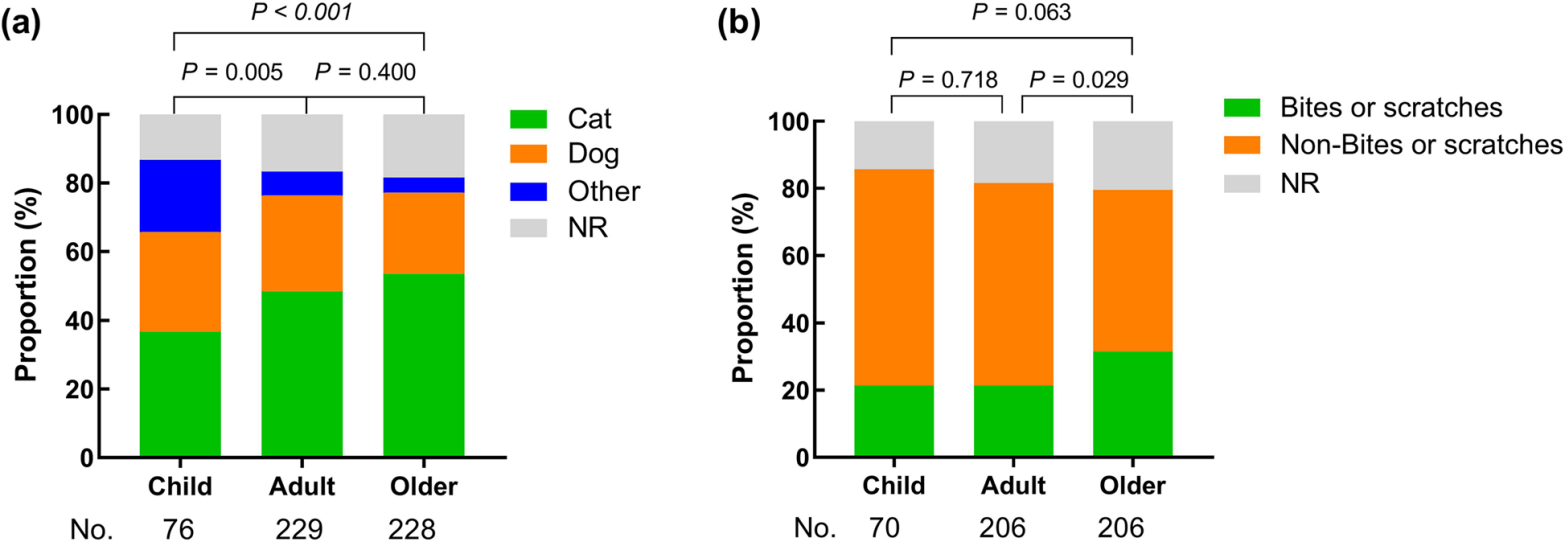
The route of infection with *P. multocida* in humans. **a** Cats and dogs are the main source of infection in three groups, meanwhile, other sources of infection are significantly high in child group; **b** *P. multocida* mainly infect the population through non-bite or scratch routes, while the number of infections by bites or scratches increase with age

The infections of *P. multocida* are mainly pet cats or dogs (Fig. 3a). Among children, cats accounted for 36.8%, dogs 28.9%; Among adults, cats 48.5%, dogs 27.9%, and elderly, cats 53.5% and dogs 23.7%. This shows that cat-related infections are more common than those from dogs. As people age, the proportion of cat-related infections increases. Additionally, cat-related infections in adults and older adults are significantly more common than in children (Comparison the sources of infection between the groups: *P* = 0.005, *P* < 0.001).

**Fig. 4:**
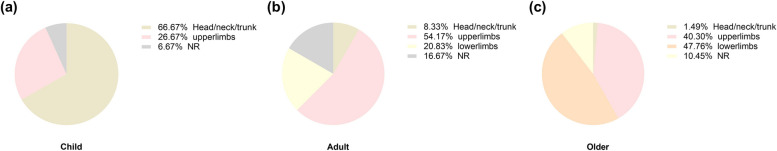
The position of animal bites or scratches in different age groups. **a** Head, neck or neck injuries are mainly in children; **b** Upper limbs injuries are mainly in adults; **c** Upper limbs and lowerlinbs injuries are mainly in the elder

P.* multocida* infection typically spreads through bites or scratches from animals (Fig. 3b). Among the children, there are 15 bites or scratches (21.4%), including cat bites or scratches 5 cases, dog bites or scratches 5 cases, weasel bites 1 case, tiger bites 2 cases, Tasmanian devil bites 1 case, and mountain lion bites 1 case; Among adults, 44 cases of bites or scratches (21.4%), including cat bites or scratches 32 cases, dog bites or scratches 12 cases, pig bites 1 cases, lion bites 1 case and tiger bites 1 case; Among the elderly, 66 cases of patients (31.5%) have been bitten or scratched, including 59 cases of cat bites and 10 cases of dog bites or scratches. Elderly people have a higher infection rate compared to children and adults (comparison of the different infection pathways between the groups: *P* = 0.063, *P* = 0.029). Based on the data on specific injury sites, among 15 cases of children who were bitten or scratched, 10 involved bites or scratches on the head, neck, or trunk, and 4 involved bites on the upper limbs. Out of 44 cases in adults, 26 involved bites on the upper limbs, 10 on the lower limbs, one person was bitten on the neck by a lion, and one person was bitten on the penis by a dog [8]; Of the 66 cases of bites or scratches in the elderly, there were 27 involving the upper limbs and 32 involving the lower limbs (Fig. 4). Regarding infections caused by *P. multocida* at different sites, injuries to the head and neck are significantly more common in children than in adults or the elderly, while in the elderly, lower limb injuries are more prevalent (Table 1). In the non-bite or scratch cases, there are some recorded infection were associated with food-producing animals or wildlife, but most are because people live with cats and dogs and have a history of contact with them or infections are caused by contact with animal secretions from the oropharynx, vertical transmission from mother to child, contamination of abdominal dialysis equipment, or inhalation of aerosols carrying *P. multocida*. Among the cases, there were also individual cases that recorded that the infected patients were slaughterhouse workers and zookeepers infected by making contact with the animals. Additionally, the source of infection is unclear in some cases, accounting for 18.7% of cases.

**Table 1:** Summarize the information about the source of infection and the site of injury from the bite and scratch cases

Group	Position	Number	Animals	Number	Avenue	Number
Child	Head/Neck/Trunk Upper limbs Lower limbs ND	10 4 0 1	Cat Dog Tiger Lion Tasmanian devil Yellow weasel	5 5 2 1 1 1	Bite Scratch Both	9 5 1
Adult	Head/Neck/Trunk Upper limbs Lower limbs ND	4^***^ 26 10 8	Cat Dog Tiger Lion Pig	32^*^ 12 1 1 1	Bite Scratch Both	23 21 0
Older	Head/Neck/Trunk Upper limbs Lower limbs ND	1^***^ 27 32^**^ 7	Cat Dog	59^***^ 10	Bite Scratch Both	47 12 7

Using the child group as a reference, *χ*^2^ test or Fisher test were performed comparing the adult or elderly group with the child group

*NR* not recorded

^*^for *P* < 0.05

^**^for *P* < 0.01

^***^for *P* < 0.001

### Main clinical features of *P. multocida* infection

**Fig. 5:**
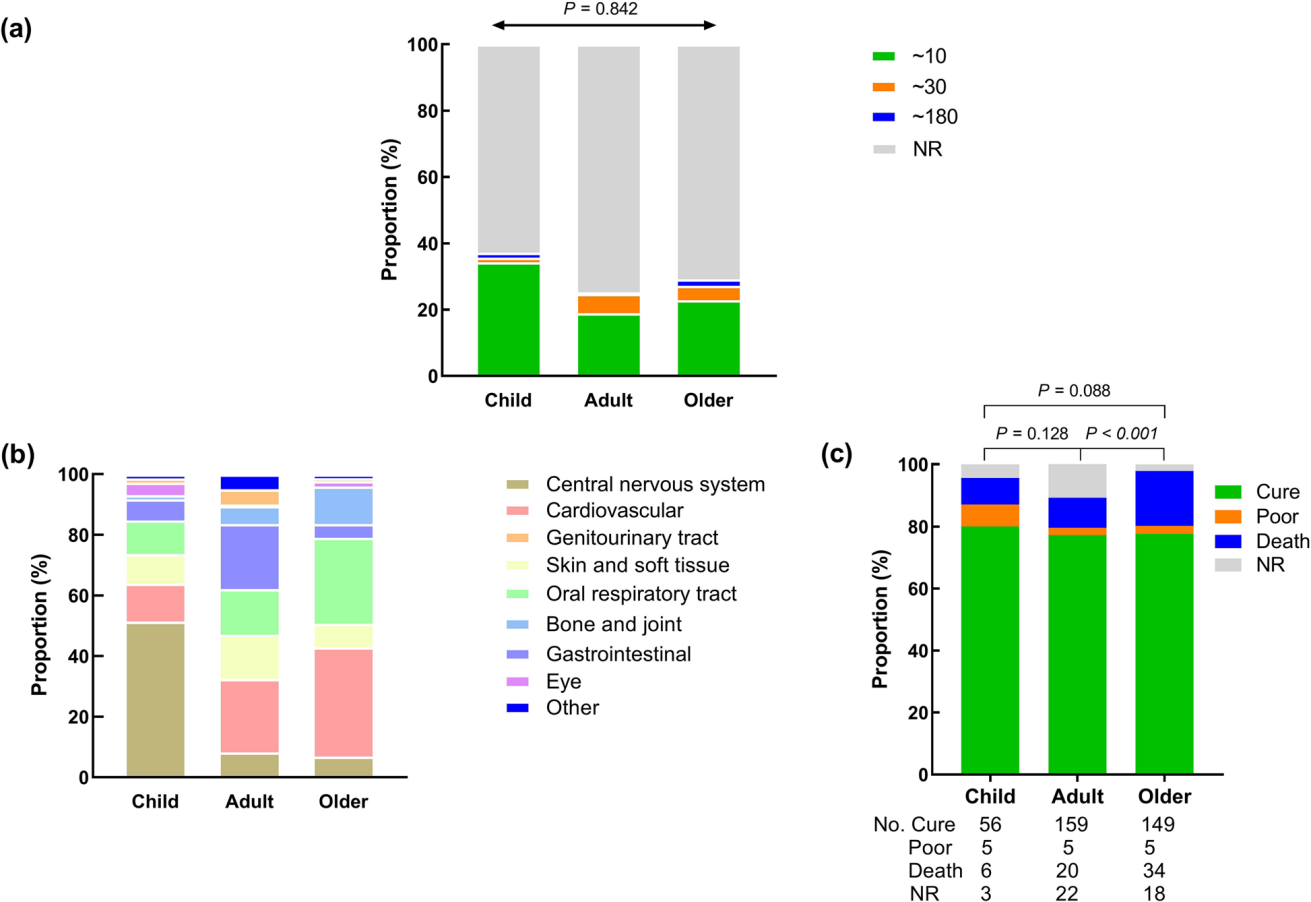
Clinical characteristics of *P. multocida* infection in humans. **a** Comparison of days of infection to onset between the groups; **b** *P. multocida* can cause systemic multi-system infection, mainly in the central nervous system, blood system, respiratory system and digestive system infection; **c** Patients with *P. multocida* infection generally have a good prognosis,clearly evident in adults. However, there is also the phenomenon of illness and disability, and it will worsen with the growth of age (comparison of the outcomes between the groups)

Regarding the infectious characteristics of *P. multocida*, most patients infected with *P. multocida* showed a disease status within 10 days, with a more significant incidence within 10 in the pediatric group. While in the adult and elderly groups, the onset time of some infected patients can be extended to one month or even longer. Among them, 33.6% (22 cases) of adults extended the onset time by one month and 1.9% (1 case) to 6 months, 15% (9 cases) of elderly groups extended the onset time by one month and 6.7% (4 cases) to 6 months (Fig. 5a).

A local wound infection from *P. multocida* usually shows symptoms like redness, pain, and pus. Besides the localized infection, this bacterium can spread to other areas and cause serious systemic infections such as bacteremia, septicemia, meningitis, brain abscesses, pneumonia, peritonitis, and intra-abdominal abscesses. At different ages, the performance of *P. multocida* infection is also different (Fig. 5b).

*P. multocida* can cause systemic multisystem infections in humans, with varying effects based on age groups. Here is a simplified summary:

- *Children*: The central nervous system (CNS) is most commonly affected, with meningitis as the primary symptom. This accounts for 51.4% of infections in this group.
- *Adults*: Infections often occur in the blood system, digestive system, respiratory system, and skin and soft tissue. These account for 24.2%, 21.5%, 15.1%, and 14.6% of adult cases, respectively. Main symptoms include septicemia, endocarditis, peritonitis, pneumonia, respiratory tract infections, epiglottitis, and wound infections.
- *Elderly*: The majority of infections in this group are hematological and respiratory system-related, with percentages of 36.1% and 28.3%, respectively. Key symptoms include septicemia, bacteremia, pneumonia, and respiratory tract infections. Some elderly patients also experience bone and joint infections.

While *P. multocida* can cause skin and soft tissue or eye infections in all age groups, there are notable differences in the systems affected among children, adults, and the elderly. The central nervous system, blood system, respiratory system, digestive system, urogenital system, and bone and joint infections show significant variations with *P*-values less than 0.05 (comparison of the clinical characteristics between tree groups).

Patients infected with *P. multocida* generally have a good outlook. However, some may not recover fully even with proper treatment, often experiencing tissue or organ damage, functional issues such as hemiplegia, vision loss, kidney damage, or, in severe cases, death. In children, a few cases of *P. multocida* infection have poor outcomes, sometimes resulting in hemiplegia or loss of vision or hearing. In adults and the elderly, the death rate from *P. multocida* infection is significantly higher, often due to age-related factors and pre-existing conditions like diabetes, high blood pressure, cirrhosis, or chronic kidney failure (Fig. 5c).

### Clinical antibiotic selection for *P. multocida* infection

**Fig. 6:**
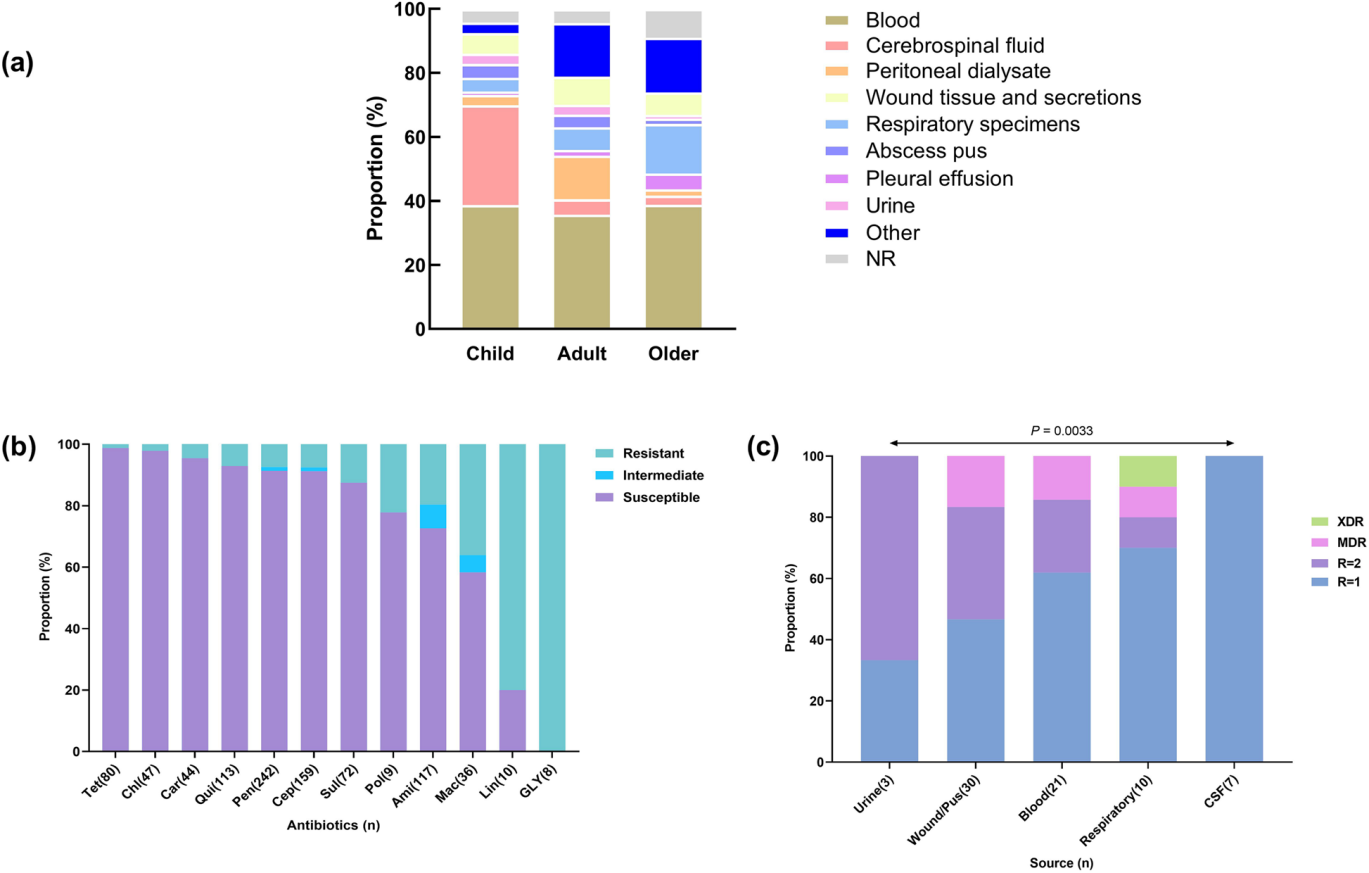
Source of *P. multocida* cultures and drug susceptibility experiments. **a** Differences in the positive rate of *P. multocida* culture in different body fluids of each group; **b** Current status of *P. multocida* resistance rates to common antibiotics; Note: Tet: *Tetracyclines*, Chl: *Chloramphenicol*, Car: *Carbapenems*, Qui: *Quinolones*, Pen: *Penicillins*, Cep: *Cephalosporins*, Sul: *Sulfonamides*, Pol:*Polypeptide antibiotics*, Ami: *Aminoglycoside*, Mac: *Macrolides*, Lin: *Lincosamides*, Gly: *Glycopeptide antibiotics*; **c** *P. multocida* resistance varies among different clinical samples. (R = 1: resistance to 1 antibiotic; R = 2: resistance to 2 antibiotics; MDR: resistance to multiple antibiotics; XDR: broad resistance)

About the diagnosis of *P. multocida*, mainly to obtain contact with animal history and culture *P. multocida* as the diagnostic basis, the main sources of culture, including blood, cerebrospinal fluid, wound secretions, oropharyngeal secretions, alveolar lavage fluid, pleural effusion, peritoneal dialysis fluid, urine, and 2 cases in which *P. multocida* was detected by genetic test of CSF or pleural water, and no clear source of the culture (Fig. 6a).

Furthermore, we count and analyze the results of drug susceptibility experiments with different positive cultures in all cases (Fig. 6b), it has been found that *P. multocida* is sensitive to *Tetracyclines*, *Chloramphenicol*, *Carbapenems*, *Quinolones*, *Penicillins* and *Cepphalosporins*, However, the resistance rate to *Macrolides*, *Lincosamides* and *Glycopeptide* antibiotics is relatively high. Of the 8 cases, *Vancomycin* are resistant.

In the study of differential resistance among different age groups, no significant difference in resistance to *P. multocida* infection was found among the age groups. While correlation studies of clinical culture sources of *P. multocida* showed significantly different antibiotic resistance among strains from different clinical samples; It’s found that CSF strains resistant to only one antibiotic, urine strains resistant to two antibiotics, blood and wound exudate strains resistant to multiple antibiotics, and respiratory strains showed extensive resistance (comparison between the amounts of antibiotics from different strains sources *P* = 0.0033 (Fig. 6c). Despite several *P. multocida* have multidrug resistance or broad resistanc, most patients can be cured after correct treatment(Table 2).

**Table 2:** Related cases of multidrug resistance or broad resistance in *P. multocida*

Case	Age	Gender	Infected animals	Drug resistance	Outcome	Reference
1	63 years	Male	An animals	Penicillins/Aminoglycosides/Polypeptide antibiotics	Death	[9]
2	52 years	Male	NR	Sulfonamides/Macrolides/Glycopeptide antibodies/Polypeptide antibiotics/Aminoglycosides	Death	[10]
3	51 years	Male	NR	Sulfonamides/Cephalosporins/Lincosamides/Aminoglycosides	Cured	[11]
4	3.5 years	Male	Cat	Penicillins/Cephalosporins/Quinolones	Cured	[12]
5	26 years	Male	NR	Penicillins/Chloramphenicol/Quinolones/Carbapenems/Cephalosporins	Cured	[13]
6	38 years	Female	Cat scratch	Penicillins/Sulfonamides/Aminoglycosides	NR	[14]
7	14 days	Male	Cat, dog	Cephalosporins/Sulfonamides/Aminoglycosides	Cured	[15]
8	47 years	Female	NR	Aminoglycosides/Cephalosporins/Penicillins/Sulfonamides	Cured	[16]
9	67 years	Male	NR	Penicillins/Cephalosporins/Aminoglycosides/Sulfonamides/Quinolones/Carbapenems	Cured	[17]

## Discussion

In *P. multocida*-infected populations, especially young children and the elderly are the most common. In the study, two peaks are found in the age of *P. multocida* infected population, namely, infants less than 1 year and the elderly in their mid-70s (Fig. 2c). On the one hand, it may be due to the large population base of this age group, on the other hand, the immune system of infants is not sound, susceptible to foreign microorganisms or pathogens, and the self-protection ability of infants is poor, vulnerable to animal bites, scratches, or licking. In the elderly population, due to the influence of basic diseases, such as hypertension, diabetes, etc., the body’s immune capacity is low, the existence of these potential factors cause the elderly to be infected with *P. multocida* when exposed to animals. There was a significant rise in infections before the age of 70, after which the sudden decline in infections may be related to life expectancy. Therefore, special attention should be paid to the problem of infection with *P. multocida* in infants and the elderly.

The main route of *P. multocida* infection is through animal vector transmission, and the main animal source of infection is cat or dog, it can also occur in some rare animal infections, such as lion [18], tiger [19–21], pig [22–24], goat [11, 25], cow [26, 27] and other animals. *P. multocida* infects humans mainly through traumatic and non-traumatic transmission. Traumatic transmission mainly refers to a local skin or soft tissue infection by biting or scratching of animals, especially in a cat or dog. Non-traumatic transmission is no direct contact with animals, but rather a wound contaminated with animal saliva or an animal licked. Our study finds that regardless of what age, *P. multocida* is often infected by cat bites or scratches, which may be related to the fact that more and more families choose to keep pet cats in today’s society and the higher carrier rate in the oropharynx of cats [1, 28]. In the child group, the probability of infection by cats or dogs is lower than that of the other two groups. We speculate that it may be because there are more children younger than 1 year, and the low expression ability and cognitive ability of children in that age are low, so there may be incomplete information record of infected animals. Therefore, in order to reduce the incidence of *P. multocida* infection, people should reduce to contact or tease emotionally unstable cats or dogs, especially stray cats and dogs, and maintain good pet hygiene management through regular cleaning and expelling parasite. Even people should avoid unnecessary intimacy with the animals when traveling in the zoo.

Human infection with *P. multocida* typically manifests within 10 days of exposure. It is even documented that inflammation at the site of a bite or scratch can occur as quickly as 24 h post-infection, generally affecting the local skin and subcutaneous tissue [4]. In addition, there are also a longer onset (1 months—6 months). We speculate that the first of factors may be the host innate immune system. Early performance is not obvious, and the pathogenic capacity of *P. multocida* is enhanced when the host is immunocompromised. Secondly, the possible factors for the relevant literature records are listed as follows:

1. It is axiomatic that adhesion to host tissues is an essential prerequisite for the establishment of infection. While *P. multocida* is able to adhere to host cells by mimicking host hyaluronic acidconstantly, which may have prolonged the infection with *P. multocida*.
2. Studies had observed encapsulated *P. multocida* within macrophages in liver sections of birds infected with the highly virulent A:1 strain VP161. Bacteria were found largely intact, with evidence of cell division, but macrophages appeared in varying stages of degeneration.We speculate that *P. multocida* can survive within human macrophages and replicate [3].

In healthy individuals, *P. multocida* typically causes redness and pain in the superficial soft tissues. However, in seriously ill individuals of healthy individuals and immunocompromised individuals, this bacterium can spread to other organs, leading to severe systemic infections. These can affect the central nervous system, cardiovascular system, respiratory system, digestive system, urinary system, and more. It may be related to the atypical symptoms of *P. multocida* infection, the difficulty of early diagnosis and the lack of knowledge of *P. multocida* among clinicians, which leads to the untimely clinical treatment and aggravates the progression of the disease.

Central nervous system infections caused by *P. multocida* are the most reported and the most common in the pediatric group; Among them, meningitis is the most common disease, most of which are occurred in infants younger than 2 months of age; Most manifestations are fever, decreased milk consumption, vomiting, and 2 cases show epileptiform manifestations [29, 30]. Meningitis has also been reported in adults or the elderly, but it is relatively rare. Childhood infections are most common partly because of children’s young age and vulnerability to licking, scratching or biting, and also because of the potential threat of the immature immune system [31].

Case reports of cardiovascular infections caused by *P. multocida* are also relatively common, including mycotic arteritis, endocarditis, septicemia, and bacteremia [32–34]. It mainly occurs in the elderly, especially combined with hypertension, diabetes, cirrhosis, rheumatoid arthritis, chronic renal insufficiency, COPD and other complications, these infected patients will develop multifunctional organ failure, leading to death [35–39]. In contrast, there are only 7 cases of *P. multocida* infection causing sepsis in children, one of which was a newborn 2 days postpartum, who died due to multifunctional organ failure and inconspicuous treatment [40].

*P. multocida* in the upper respiratory tract infection can appear sinusitis, epiglottis, tonsillitis [41–43]; But in the lower respiratory tract infection, the elderly infection is mainly manifested as pneumonia, pulmonary granuloma, empyema, lung abscess, tracheobronchitis and a case of squamous lung carcinoma patient Combined with *P. multocida* infection [26, 44–47]. In children, the infection is relatively rare, only 6 cases of pneumonia, 1 case of empyema [48], 1 case of lung abscess [49], etc.; Inside one case of neonatal pneumonia died after postpartum treatment, the mother of the children had a history of prenatal esposure to cats and *P. multocida* was cultured in both the child’s nasopharyngeal secretion and the maternal cervical-vaginal secretions [50].

Peritoneal dialysis patients are at risk of getting peritonitis caused by *P. multocida*. This infection can lead to symptoms like fever, abdominal pain, and cloudy dialysis fluid. The infection often spreads when a cat licks or bites the dialysis tube, and many of these patients had close contact with cats before beginning dialysis [51, 52]. Peritonitis caused by *P. multocida* can occur at any age but is more commonly seen in adult patients undergoing peritoneal dialysis and only three cases have been reported in children [53–55]. In addition to common infections, in children, *P. multocida* infection can cause corneal ulcer [43], conjunctivitis [56], etc. Adults and the elderly have graft infection due to a history of contact with cats or dogs, including prosthetic joint infection, breast graft infection [57–60], vascular graft infection. Furthermore, there was an elderly patient after kidney transplantation, because the cat licking the leg ulcer caused sepsis, which finally led to concurrent cardiopulmonary failure and died [61]. Urinary tract infections caused by *P. multocida* are relatively rare, and individual case had been reported in children, adults and the elderly [62–64]. Regarding the study of *P. multocida* antimicrobial agents, we have found that the resistance rate of *P. multocida is increasing,* it may be due to individual differences and the excessive overuse of antibiotics.

Moreover, the strains of different clinical samples have different resistance results to antibiotics. Firstly the interpretation of this different result may be due to the different plasmid genomes of *P. multocida.* The most frequently used molecular typing methods for *P. multocida* are capsular genotyping, LPS genotyping, MLST, and virulence genotyping based on the detection of different virulence gene profiles. However, its drug resistance is mainly related to the plasmid genomes, the different genotyped strains all contain different plasmid genomes, such as: pIG1, pS298D, pB1018, pB1006, pB1005, pB1000, pOV, pJR1, pJR2, pCCK1900 and pCCK411, etc., which carry antibiotic resistance genes. A β-lactam resistance gene was identified on plasmid pB1000, *Sulfonamides* and *Streptomycin* resistance genes on pB1005 and pB1006, and a *Tetracycline* resistance gene was identified on pB1006. Plasmid pJR1 contains resistance genes to *Sulfonamide* drugs, *Tetracycline* and *Chloramphenicol*, while plasmid pJR2 carries resistance genes against Streptomycin and *Spectinomycin* as well as *Ampicillin* and *Carboxylpenicillin.* In addition, *P. multocida* leads to different virulence manifestations and outcomes by encoding different virulence genes,like fimbriae and other adhesins, toxin, iron acquisition proteins, sialidases, hyaluronidase, outer membrane proteins, and superoxide dismutase, Such as: 1) Type A strains carrying the adhesin gene are more likely to colonize the respiratory mucosa, causing pneumonia or sepsis. 2) In the iron uptake system, the HgbA gene helps the strain to obtain iron from hemoglobin, promoting its proliferation in the blood, leading to acute sepsis. 3) The G-protein-deamidating toxin of *P. multocida* can lead to progressive atrophic rhinitis and dermonecrosis [65]. Next may be related to the differences in the concentration of antimicrobial agents at different infection sites. For example, in the central nervous system tissue, the third and fourth generation of *Cephalosporins*, *Aztreonam*, *Carbapenems*, *Sulfonamides*, *Quinolones*, *Vancomycin*, *Rifampin* and *Isoniazid* and other drugs of the blood–brain barrier penetration is high, the concentration in the cerebrospinal fluid is higher, so the drug resistance is low. In the abdominal tissue, *Penicillins*, *Cephalosporins*, β-lactamase inhibitor compounds, *Quinolones*, *Carbapenems*, *Metronidazole* and *Vancomycin* had high concentrations in ascites. In the blood flow, when the drug apparent volume is smaller, the protein binding rate is higher. It means that slow drug penetration into tissues, high blood concentration and a long time, the better the effect on blood flow infection,such as *Penicillin*, *Cephalosporins*, β-lactamase inhibitor compounds, *Carbapenems*, *Vancomycin* and *Daptomycin*, etc. In the skin and skin soft tissues, generally lipophilic antibacterial drugs often have higher skin and skin soft tissue permeability than hydrophilic antibacterial drugs, such as *Gatifloxacin*, *Linezolid* and *Levofloxacin* and other drugs. In the distribution of the urinary system, Most *Quinolones* and β-lactams have high urinary concentrations and can be used for the treatment of upper urinary and lower urinary tract infections. While in respiratory tissues, *Macrolides*, *Quinolones*, *Tigecycline*, and *Linezolid* had significantly higher concentrations in the respiratory epithelial lining fluid [66–69]. Besides, for the animal experiments of *P. multocida*, some studies have explained the differences in the colonization of the lung, liver, thymus, spleen, heart and kidney of rabbits, which finding that the lung and thymus had the highest number of *P. multocida,* but no *P. multocida* were detected in kidney [70]. But there is still no study on human infection. We reasoned that similar or different results might also exist for the content of *P. multocida* in different tissues in humans and it is worth our further study.

Therefore, for human infections with *P. multocida*, *Penicillin is* as the first-line medication for treatment before susceptibility testing, because it is safe, effective, easy access to cerebrospinal fluid and other tissues and cheap. However, β-lactamase-resistant strains were isolated in human respiratory specimens [71], and the presence of multiple drug-resistant strains. Combining the relevant literature and our statistical results, we suggest that second- and third-generation *Cephalosporins, Fluoroquinolones,* and *Tetracyclines* are recommended for treatment [72]. Finally, it is recommended that antibiotic selection should combine the clinical pharmacology of bacterial antibiotics and susceptibility testing of specific strains and choose the appropriate antibiotic regimen and antibiotic drugs dosage, to avoid drug abuse.

Research indicates that out of 265 non-bite cases, 22 cases were dead cases(8.3%), 209 cases were cured cases(78.9%), 8 cases had poor prognosis(3%) and 26 cases were unrecorded(9.8%). While out of 127 bite or scratch cases, 15 cases were dead cases(11.8%), 98 cases were cured cases(77.2%), 5 cases had poor prognosis(3.9%) and 9 cases were unrecorded(7.1%). The overall prognosis of non-bite infection is good and consistent with the conclusions of most studies [73]. And, in terms of the prognosis of *P. multocida* infection, most of the 60 dead cases caused severe infections such as sepsis and central nervous system infections, especially in adults and the elderly. While in multi-resistant patients, some resistant strains may acquire resistance through genetic mutations, but with decreased virulence, resulting in low actual fatality rate, which requires more data support. Thus, whether about the treatment of bites and scratches or non-bites and scratches, the clinical treatment requires attention to the immune function status of patients, site of infection, and timeliness of diagnosis, which can significantly affect the outcome. For instance, in elderly individuals with pre-existing health problems or compromised immune systems, these infections often lead to death due to complications involving multi-organ failure [74]. Therefore, in treating *P. multocida* infections, it is crucial to actively monitor patients’ vital signs, assess the severity of their condition, and maintain stable blood glucose and blood pressure, especially in elderly patients. For those with severe infections, consider transferring them to the intensive care unit if needed. Active management of underlying health conditions and ongoing evaluation of disease progression are particularly important to ensure effective treatment and improve prognosis.

However, this study has several limitations, including the broad time range and geographical scope of data collection, potential ethnic disparities, and information bias. To address these issues, future research could focus on conducting a retrospective study with a larger sample from a specific region and population over a defined period. This would allow for a more comprehensive examination of the clinical characteristics of *P. multocida* infections across different age groups. In addition, in areas with limited healthcare resources, there may be insufficient detection capacity. Therefore, the reported cases of *P. multocida* to public health authorities may be lower than the actual number of cases. Meanwhile, the lack of incidence and prevalence estimates of *P. multocida* in different age populations our study limits our ability to draw precise and comprehensive conclusions about the global burden of *P. multocida.*

## Conclusion

The infection of *P. multocida* is universal in age, but it is more common in people with low immunity, and the clinical characteristics and prognosis are obviously heterogeneous in different ages. The infection route of *P. multocida* is mainly through the bites or scratches of cats or dogs, and with the increase of cats and dogs, the infection rate of *P. multocida* is gradually increased. The results of drug susceptibility test showed that *P. multocida* was sensitive to penicillin antibiotics. In addition, it should be noted that in special groups such as peritoneal dialysis, special attention should be paid to the health and safety of the use of instruments.

## Supplementary Information

Supplementary Material 1.

## Data Availability

The datasets used during the current study are Collected in the publicly available literature.
