# Supplementary material for: Pasteurella multocida infection: a differential retrospective study of 482 cases of P. multocida infection in patient of different ages
Source: BMC Infect Dis. 2025 Mar 4;25:313. doi: 10.1186/s12879-025-10711-1 (PMC11881477; doi:10.1186/s12879-025-10711-1)
Supplement: Supplementary file 1 — Supplementary Material 1. [file 12879_2025_10711_MOESM1_ESM.zip › Supplementary Table and the reference_ESM.docx]

**Supplementary Table. Related cases of drug resistance in *P .multocida*.**

| **Case** | **Age** | **Gender** | **Infected animals** | **Drug resistance** | **Outcome** | **Reference** |
| --- | --- | --- | --- | --- | --- | --- |
| 1 | Term infant | Male | Cat, dog | Aminoglycosides | Cured | [1] |
| 2 | Neonate | Female | History of maternal contact with the dog | Cephalosporins | Cured | [2] |
| 3 | 30 days | NR | Cat | Aminoglycosides | Cured | [3] |
| 4 | 2 days | Male | History of maternal contact with the cat | Macrolides | Cured | [4] |
| 5 | 55 years | Female | Cat bite dialysis tube | Penicillins/Cephalosporins | Cured | [5] |
| 6 | 54 years | Male | Cat bite dialysis tube | Glycopeptide antibodies | Cured | [6] |
| 7 | 42 years | Female | Cat bite dialysis tube,dog | Glycopeptide antibodies | Cured | [7] |
| 8 | 36 years | Female | Cat licking | Macrolides | NR | [8] |
| 9 | 46 years | Female | Cat | Glycopeptide antibodies | Cured | [9] |
| 10 | 75 years | Male | Dog | Penicillins(Ampicillin) | Death | [10] |
| 11 | 46 years | Male | NR | Aminoglycosides | Cured | [11] |
| 12 | 76 years | Female | NR | Macrolides | Death | [12] |
| 13 | 87 years | Male | Cat, dog | Aminoglycosides/Macrolides | Cured | [13] |
| 14 | 51 years | Male | Cat scratch | Aminoglycosides | Death | [14] |
| 15 | 62 years | Male | NR | SulFemaleonamides | Death | [14] |
| 16 | 89 years | Female | Cat scratch | Macrolides | Cured | [15] |
| 17 | 20 months | Male | Cat | Quinolones/SulFemaleonamides | Cured | [16] |
| 18 | 13 years | Male | Cat bite and scratch | Glycopeptide antibodies/Lincosamides | Cured | [17] |
| 19 | 15 years | Male | Cat | Penicillins | Poor | [18] |
| 20 | 84 years | Male | NR | Lincosamides | Cured | [19] |
| 21 | 82 years | Male | NR | Lincosamides | Death | [19] |
| 22 | 14 years | Female | NR | Aminoglycosides | Poor | [20] |
| 23 | 75 years | Female | Cat | Penicillins | Cured | [21] |
| 24 | 27 years | Male | NR | Quinolones/Tetracyclines | Cured | [22] |
| 25 | 67 years | Female | Cat | Macrolides | Cured | [23] |
| 26 | 50 years | Male | NR | Penicillins | Cured | [24] |
| 27 | 65 years | Male | NR | Macrolides | Cured | [25] |
| 28 | 54 years | Male | Cat bite | Penicillins | Death | [26] |
| 29 | 69 years | Female | Dog | Aminoglycosides | Death | [27] |
| 30 | 45 years | Male | Dog bite | Penicillins | NR | [28] |
| 31 | 31 years | Female | Dog licking | Lincosamides | Cured | [29] |
| 32 | 18 years | Male | Animals | Aminoglycosides | Cured | [30] |
| 33 | 60 years | Male | NR | Aminoglycosides | NR | [31] |
| 34 | 57 years | Male | Dog licking | Macrolides/Lincosamides | Cured | [32] |
| 35 | 66 years | Female | Cat | Penicillins/Cephalosporins | NR | [33] |
| 36 | 52 years | Male | Dog bite | Aminoglycosides | Death | [34] |
| 37 | 63 years | Female | Cat | SulFemaleonamides/Aminoglycosides | Cured | [35] |
| 38 | 48 years | Female | NR | Aminoglycosides/Lincosamides | Cured | [36] |
| 39 | 80 years | Female | NR | Quinolones | NR | [37] |
| 40 | 64 years | Female | Cat bite | Penicillins | Cured | [38] |
| 41 | 73 years | Male | NR | Penicillins/Cephalosporins | Cured | [39] |
| 42 | 44 years | Male | NR | Macrolides/Quinolones | Death | [40] |
| 43 | 68 years | Male | Cat, dog | Aminoglycosides/Quinolones | Cured | [41] |
| 44 | 52 years | Female | NR | Aminoglycosides/Macrolides | Cured | [42] |
| 45 | 76 years | Male | Cat bite | Penicillins | Cured | [43] |
| 46 | 16 years | Female | NR | Penicillins/Cephalosporins | Cured | [44] |
| 47 | NR | NR | NR | Glycopeptide antibodies | NR | [45] |
| 48 | 54 years | Female | NR | Aminoglycosides/Macrolides | NR | [46] |
| 49 | 63 years | Male | An animal | Penicillins/Aminoglycosides/Polypeptide antibiotics | Death | [47] |
| 50 | 52 years | Male | NR | SulFemaleonamides/Macrolides/  Glycopeptide antibodies/Polypeptide antibiotics/Aminoglycosides | Death | [48] |
| 51 | 51 years | Male | NR | SulFemaleonamides/Cephalosporins/Lincosamides/Aminoglycosides | Cured | [49] |
| 52 | 3.5 years | Male | Cat | Penicillins/Cephalosporins/Quinolones | Cured | [50] |
| 53 | 26 years | Male | NR | Penicillins/Chloramphenicol/Quinolones/Carbapenems/Cephalosporins | Cured | [51] |
| 54 | 38 years | Female | Cat scratch | Penicillins/SulFemaleonamides/Aminoglycosides | NR | [52] |
| 55 | 14 days | Male | Cat, dog | Cephalosporins/SulFemaleonamides/Aminoglycosides | Cured | [53] |
| 56 | 47 years | Female | NR | Aminoglycosides/Cephalosporins/Penicillins/SulFemaleonamides | Cured | [54] |
| 57 | 67 years | Male | NR | Penicillins/Cephalosporins/Aminoglycosides/SulFemaleonamides/Quinolones/Carbapenems | Cured | [44] |
| 58 | NR | NR | NR | Aminoglycosides/Macrolides/Cephalosporins/Glycopeptide antibodies/Lincosamides | NR | [55] |

1. Zaramella P, Zamorani E, Freato F, Cattai M, Meloni GA. Neonatal meningitis due to a vertical transmission of Pasteurella multocida. Pediatr Int. 1999 Jun;41(3):307-310.
2. Pace D, Attard-Montalto S. Quest for the diagnosis. Case 1: a neonatal zoonosis. Neonatal Pasteurella multocida septicaemia. Acta Paediatr. 2008 Feb;97(2):250-252.
3. P Repice;E Neter.Pasteurella multocida meningitis in an infant with recovery[J].The Journal of pediatrics,1975,Vol.86(1): 91-93
4. Hillery S, Reiss-Levy EA, Browne C, Au T, Lemmon J. Pasteurella multocida meningitis in a two-day old neonate. Scand J Infect Dis. 1993;25(5):655-658.
5. Paul RV, Rostand SG. Cat-bite peritonitis: Pasteurella multocida peritonitis following feline contamination of peritoneal dialysis tubing. Am J Kidney Dis. 1987 Oct;10(4):318-319.
6. London RD, Bottone EJ. Pasteurella multocida: zoonotic cause of peritonitis in a patient undergoing peritoneal dialysis. Am J Med. 1991 Aug;91(2):202-204.
7. Uribarri J, Bottone EJ, London RD. Pasteurella multocida peritonitis: are peritoneal dialysis patients on cyclers at increased risk? Perit Dial Int. 1996 Nov-Dec;16(6):648-649.
8. Mugambi SM, Ullian ME. Bacteremia, sepsis, and peritonitis with Pasteurella multocida in a peritoneal dialysis patient. Perit Dial Int. 2010 May-Jun;30(3):381-383.
9. Olea T, Hevia C, Bajo MA, del Peso G, Selgas R. Peritonitis por pasteurella multocida y Candida albicans [Pasteurella multocida and Candida albicans peritonitis]. Nefrologia. 2006;26(1):136-138.
10. Jogani SN, Subedi R, Chopra A, Judson MA. Pasteurella multocida pleural effusion: A case report and review of literature. Respir Med Case Rep. 2016 Jul 21;19:68-70.
11. OLSEN AM, NEEDHAM GM. Pasteurella multocida in suppurative diseases of the respiratory tract. Am J Med Sci. 1952 Jul;224(1):77-81.
12. HORNE WI, BERLYNE GM. Empyema caused by Pasteurella septica. Br Med J. 1958 Oct 11;2(5101):896.
13. Kofteridis DP, Christofaki M, Mantadakis E, Maraki S, Drygiannakis I, Papadakis JA, Samonis G. Bacteremic community-acquired pneumonia due to Pasteurella multocida. Int J Infect Dis. 2009 May;13(3):e81-83.
14. Gerding DN, Khan MY, Ewing JW, Hall WH. Pasteurella multocida peritonitis in hepatic cirrhosis with ascites. Gastroenterology. 1976 Mar;70(3):413-415.
15. Kumar A, Devlin HR, Vellend H. Pasteurella multocida meningitis in an adult: case report and review. Rev Infect Dis. 1990 May-Jun;12(3):440-448.
16. Goussard P, Gie RP, Steyn F, Rossouw GJ, Kling S. Pasteurella multocida lung and liver abscess in an immune-competent child. Pediatr Pulmonol. 2006 Mar;41(3):275-278.
17. Hutcheson KA, Magbalon M. Periocular abscess and cellulitis from Pasteurella multocida in a healthy child. Am J Ophthalmol. 1999 Oct;128(4):514-515.
18. Baliah T, Neter E. Pasteurella multocida infection of urinary tract in patient with ileal loop. Urology. 1977 Mar;9(3):294-5. doi: 10.1016/0090-4295(77)90350-90358.
19. Rose HD, Mathai G. Acute Pasteurella multocida pneumonia. Br J Dis Chest. 1977 Apr;71(2):123-126.
20. Larsen TE, Harris L, Holden FA. Isolation of Pasteurella multocida from an otogenic cerebellar abscess. Can Med Assoc J. 1969 Nov 15;101(10):114-115.
21. Lion C, Lozniewski A, Rosner V, Weber M. Lung abscess due to beta-lactamase-producing Pasteurella multocida. Clin Infect Dis. 1999 Nov;29(5):1345-1346.
22. Maleb A, Elmalki J, Bouayadi O, Ben Lahlou Y, Frikh M, Abdeljaouad N, Lemnouer A, Yacoubi H, Elouennass M. Serious phlegmonous lesion of the hand following an injury by vegetal thorn: Never forget Pasteurella multocida! Trauma Case Rep. 2017 Nov 27;13:18-21.
23. Katechakis N, Maraki S, Dramitinou I, Marolachaki E, Koutla C, Ioannidou E. An unusual case of Pasteurella multocida bacteremic meningitis. J Infect Public Health. 2019 Jan-Feb;12(1):95-96.
24. Branch J, Kakutani T, Kuroda S, Shiba Y, Kitagawa I. Pasteurella multocida Infective Endocarditis: A Possible Link with Primary Upper Respiratory Tract Infection. Intern Med. 2015;54(24):3225-3231.
25. Steyer BJ, Sobonya RE. Pasteurella multocida lung abscess. A case report and review of the literature. Arch Intern Med. 1984 May;144(5):1081-1082.
26. Jones AG, Lockton JA. Fatal Pasteurella multocida septicaemia following a cat bite in a man without liver disease. J Infect. 1987 Nov;15(3):229-235.
27. Migliore E, Serraino C, Brignone C, Ferrigno D, Cardellicchio A, Pomero F, Castagna E, Osenda M, Fenoglio L. Pasteurella multocida infection in a cirrhotic patient: case report, microbiological aspects and a review of literature. Adv Med Sci. 2009;54(1):109-112.
28. Naas T, Benaoudia F, Lebrun L, Nordmann P. Molecular identification of TEM-1 beta-lactamase in a Pasteurella multocida isolate of human origin. Eur J Clin Microbiol Infect Dis. 2001 Mar;20(3):210-213.
29. Schmulewitz L, Chandesris MO, Mainardi JL, Poirée S, Viard JP, Lecuit M, Mamzer-Bruneel MF, Lortholary O. Invasive Pasteurella multocida sinusitis in a renal transplant patient. Transpl Infect Dis. 2008 Jun;10(3):206-208.
30. Thomas C, Quinio P, Tande D. Pasteurellose pulmonaire chez un jeune polytraumatisé [Pulmonary pasteurellosis in a young patient with multiple trauma]. Ann Fr Anesth Reanim. 1992;11(1):103-104.
31. Lee WS, Chen FL, Wang CH, Ou TY, Lin YH, Jean SS. Community-acquired bacteremic pneumonia due to Pasteurella multocida subspecies multocida in a patient with poor-control diabetes mellitus. J Microbiol Immunol Infect. 2019 Feb;52(1):163-164.
32. Maraki S, Kastanis G, Stafylaki D, Masunt S, Kapsetakis P, Scoulica E. Pasteurella multocida wound infection transmitted by a pet dog. Germs. 2018 Dec 3;8(4):214-217.
33. Larnè D, Ceccarelli M, Condorelli F, Rullo EV, Nunnari G, Pellicanò GF. Bacteremic meningitis due to Pasteurella multocida resistant to first line antibiotic therapy. Infect Dis Rep. 2018 Sep 5;10(2):7632.
34. Borges L, Oliveira N, Cássio I, Costa H. Sepsis-induced purpura fulminans caused by Pasteurella multocida. BMJ Case Rep. 2014 Feb 19;2014:bcr2013202441.
35. Blomgren G, Malmborg AS. Late postoperative infection caused by Pasteurella multocida. Scand J Infect Dis. 1980;12(2):153-154.
36. Chong Y, Lee HJ, Lee SY, Jahng JS, Yang KH. Pasteurella multocida infection of the calf in a patient who had moxa cautery treatment for degenerative arthritis. Yonsei Med J. 1982;23(1):65-70.
37. Hiura K, Yamada H, Yamaguchi T, Katoh O, Nagasawa Z. [A case of Pasteurella multocida infection in bronchiectasis]. Kansenshogaku Zasshi. 1990 Jul;64(7):866-870.
38. Williams RA, Fincham WJ. Septic arthritis due to Pasteurella multocida complicating rheumatoid arthritis. Ann Rheum Dis. 1979 Aug;38(4):394-395.
39. Chen Mindong, Zhu Yanyan, Xu Yiwei, Liu Yuting, Wang Qiong, Wang Yao. A case report of hemodialysis catheter-related sepsis caused by Pasteurella multocida [J]. Journal of Fujian Medical University, 2012, (5): 370
40. Yu Aiyu, Wang Yan, Zhang Kailing, et al. A case of surgical incision infection caused by Pasteurella multocida [J]. Chinese Journal of Hospital Infectious Diseases, 2008, (08): 1142.
41. Wang Mengmeng, Pan Meier, Wang Jingquan, et al. A case of leprosy ulcer accompanied by Pasteurella multocida infection [J]. Dermatology and Venereal Diseases, 2007, (02): 63.
42. Ma Yanchun, Wei Jianjun, Zhang Naihang. A case of pleurisy complicated by urinary tract infection caused by Pasteurella multocida [J]. Journal of Jining Medical College, 1999, (04): 25.
43. XU Yuanping. A case of severe Pasteurella multocida infection [J]. Journal of Hubei University of Science and Technology (Medical Edition), 2017, 31 (03): 273.
44. Ge Fengyuan, Yuan Yuan. Analysis of pathogenicity and drug resistance of two Pasteurella multocida strains [J]. Journal of Industrial Enterprise Medical, 2011, 24 (05): 8-9.
45. Li Yuping. Isolation and identification of 6 Pasteurella multocida strains [J]. Shanghai Journal of Medical Laboratory, 1997, (04): 232.
46. Ma Yanchun. Simultaneous isolation of Pasteurella multocida from pleural fluid and urine [J]. Shanghai Journal of Medical Laboratory, 1998, (04): 243.
47. Schmidt EC, Truitt LV, Koch ML. Pulmonary abscess with empyema caused by Pasteurella multocida. Report of a fatal case. Am J Clin Pathol. 1970 Nov;54(5):733-736.
48. Maneche, H.C. and H.J. TOLL, Pulmonary Cavitation and Massive Hemorrhang Caused by Pasteurella multocida. Report of a Case. N Engl J Med. 1964 Sep 3;271:491-494.
49. Al-Ghonaim MA, Abba AA, Al-Nozha M. Endocarditis caused by Pasteurella multocida. Ann Saudi Med. 2006 Mar-Apr;26(2):147-149.
50. Yuanping Xu. A Case of Severe Pasteurella multocida Infection. Journal of Hubei University of Science and Technology (Medical Edition).2017; 31(03):273.
51. Wang Huafen, Zhang Chengwei. A case of Open Tarsal Fracture Complicated with Pasteurella multocida Infection. Chinese Journal of Orthopaedics. 2000;7(5):433.
52. Xia Xiangyun, Sun Enhua. Simultaneous Isolation of Pasteurella multocida and Salmonella Paratyphi A from Animal Bite Wound Secretions: A Case Report. Journal of North China Coal Medical College. 2004;6(5):596-596.
53. Wang Jianyue. Identification and Drug Resistance Analysis of Pasteurella multocida in Neonates. Shaanxi Medical Journal. 2016;45(23):2843.
54. Wang Chengying, Chang Ji, Song Quanchun. Isolation of Pasteurella multocida from Cervical Secretions. Chinese Journal of Medical Laboratory. 2004;5(4):365-365.
55. Wang Hongqi, Wei Yanling. Pasteurella multocida and human infection [J]. Chinese Journal of Zoonoses, 1993, (01): 54-55.
